# Domain-Adaptive Prototype-Recalibrated Network with Transductive Learning Paradigm for Intelligent Fault Diagnosis under Various Limited Data Conditions

**DOI:** 10.3390/s22176535

**Published:** 2022-08-30

**Authors:** Jiachen Kuang, Tangfei Tao, Qingqiang Wu, Chengcheng Han, Fan Wei, Shengchao Chen, Wenjie Zhou, Cong Yan, Guanghua Xu

**Affiliations:** 1 School of Mechanical Engineering, Xi’an Jiaotong University, Xi’an 710049, China; 2 State Key Laboratory for Manufacturing Systems Engineering, Xi’an Jiaotong University, Xi’an 710049, China; 3 Key Laboratory of Education Ministry for Modern Design & Rotor-Bearing System, Xi’an 710049, China

**Keywords:** intelligent fault diagnosis, transductive domain adaptation, prototype recalibration strategy, limited data conditions

## Abstract

In real industrial scenarios, intelligent fault diagnosis based on data-driven methods has been widely researched in the past decade. However, data scarcity is widespread in fault diagnosis tasks owning to the difficulties in collecting adequate data. As a result, there is an increasing demand for both researchers and engineers for fault identification with scarce data. To address this issue, an innovative domain-adaptive prototype-recalibrated network (DAPRN) based on a transductive learning paradigm and prototype recalibration strategy (PRS) is proposed, which has the potential to promote the generalization ability from the source domain to target domain in a few-shot fault diagnosis. Within this scheme, the DAPRN is composed of a feature extractor, a domain discriminator, and a label predictor. Concretely, the feature extractor is jointly optimized by the minimization of few-shot classification loss and the maximization of domain-discriminative loss. The cosine similarity-based label predictor, which is promoted by the PRS, is exploited to avoid the bias of naïve prototypes in the metric space and recognize the health conditions of machinery in the meta-testing process. The efficacy and advantage of DAPRN are validated by extensive experiments on bearing and gearbox datasets compared with seven popular and well-established few-shot fault diagnosis methods. In practical application, the proposed DAPRN is expected to solve more challenging few-shot fault diagnosis scenarios and facilitate practical fault identification problems in modern manufacturing.

## 1. Introduction

With the advancement of the Industrial Internet of Things (IIoT), modern manufacturing based on the complex industrial system is progressing towards high-level precision and speed, accompanied by high demand for reliability and health management [1]. In engineering practice, any unexpected failure of large industrial mechanical equipment may lead to serious casualties and economic losses [2,3]. Hence, accurate fault diagnosis, which is of great importance for equipment health management, has received more and more attention from both academia and industry.

Recently, a lot of intelligent fault diagnosis methods based on deep learning (DL) models which can automatically recognize health conditions of machinery in an end-to-end manner have made major advances, such as sparse auto-encoder (SAE) [4], convolutional neural network (CNN) [5,6], generative adversarial network (GAN) [7,8], graph neural network (GNN) [9], long short-term memory network (LSTM) [10], and deep belief network (DBN) [11]. In general, DL-based fault diagnosis models directly learn the mapping between the training dataset with available labels and recognize the unseen health conditions in the testing dataset, and the training and testing datasets are implicitly in the same distribution. To solve the cross-domain fault identification, transfer learning (TL), which leverages learned diagnostic knowledge from similar and relative tasks in the source domain and identifies the health conditions of the target domain, has been developed in recent years [12,13,14,15,16,17,18]. In the TL scheme, the health conditions of source and target domains are generally the same, except for some partial TL methods [19,20,21,22] and open set TL methods [23,24]. Moreover, the TL scheme is also data-greedy, similar to the DL scheme. Based on the above analysis, both traditional DL and TL have been successfully implemented in data-intensive applications but they are usually hampered when data are limited. In the field of fault diagnosis, the original data are collected and classified by faulty types and operating conditions. In modern production, it is apparent that machinery does not always work under high load and speed, except in emergencies. Additionally, the fault identification and health management system does not allow machines to work in faulty states; only limited original data with supervised labels are collected in practice. Unfortunately, these diagnostic tasks with data-scarce cases are common in real-world modern machinery. Consequently, intelligent fault diagnosis with scarce data is a scenario with ever-increasing demand in practical engineering.

Few-shot learning (FSL) [16] is a promising tool to solve the above challenge, which aims to train a classifier to diagnose unseen health conditions during the training process with limited labeled data. Motivated by strong generalization ability of humans to perform even one-shot classification, FSL is widely explored in several fields [17], for instance, image classification [18], intrusion detection [19], disease prediction [20], and fault diagnosis [21]. To recognize the health conditions of bearings, Zhang et al. [22] proposed a few-shot diagnosis model based on the Siamese neural network (SiameseNet), which learns the similarity-based information in feature space by inputting sample pairs of the same or different classes. Ren et al. [1] presented a novel few-shot model composed of an auto-encoder and capsule network, which shows high performance on few-show fault diagnosis tasks. Inspired by the model-agnostic meta-learning (MAML), a few-shot learning framework whose fault classifier is trained with limited target data is proposed for bearing fault diagnosis [23], the MAML-based framework is further validated by recognizing new bearing damages of a public dataset. Combined with supervised domain adaptation and prototype learning, a Siamese architecture is proposed to learn the domain-invariant space for bearing fault identification with limited data [24]. As a variation of the metric-based meta-learning method, Wang et al. [25] presented a reinforced relation network for rolling element bearing fault identification with few-shot samples. Based on the deep metric of relation network (RelationNet) [26], Wu et al. [27] proposed a unified convolutional neural network for few-shot diagnosis tasks for machinery. Further, a metric-based meta-learning model with global supervised training and episodic training in feature space, which is extended from the matching network (MatchingNet) [28] and prototypical network (ProtoNet) [29], is proposed for fault diagnosis of machinery under various limited data conditions [21].

Through the systematic literature review, it can be concluded that these existing networks with few-shot generalization ability have made great progress in fault diagnosis with limited data in real industrial scenarios. Two main branches of the FSL, i.e., optimization-based meta-learning [23,30] and metric-based meta-learning [21,24], have been applied in the intelligent fault diagnosis of machinery. Moreover, the metric-based meta-learning methods show great potential in the field of few-shot fault diagnosis. Despite the various design of network architecture in metric-based meta-learning methods, these methods are composed of a network-based feature extractor for high-level representation extraction and a similarity-based classifier for fault diagnosis with unseen health conditions. During the meta-learning phase, the episodic training method is applied to randomly sample mini-batches as episodes to mimic the few-shot tasks in the source dataset with base classes and generalize the diagnostic knowledge to facilitate few-shot fault diagnosis tasks in the target dataset with novel classes. Nonetheless, there are still two drawbacks to these aforementioned studies. (1) Note that most existing few-shot diagnosis methods make the implicit hypothesis that the source and target data are from the same distribution. However, this hypothesis is not valid due to the huge difference in the working conditions of machinery in real-industrial scenarios. (2) Metric-based meta-learning methods require identifying faults of machinery with limited labeled samples in practical engineering. However, the narrow-size target distribution, which relies heavily on scarce data from the target domain, tends to train a biased similarity-based classifier.

To solve the above-mentioned issues, a novel FSL method named domain-adaptive prototype-recalibrated network (DAPRN) with transductive learning paradigm and prototype recalibration strategy (PRS) is proposed for intelligent fault diagnosis of machinery under various limited data conditions. The proposed DAPRN, which is inspired by the idea of ProtoNet, is composed of a feature extractor, a domain discriminator, and a cosine similarity-based label predictor. Integrating the domain adaptation into the meta-training process, the feature extractor of DAPRN learns a domain-invariant representation space between the source and target domains through joint optimization of minimizing the few-shot classification loss and maximizing the domain-discriminative loss. The prototypes, which denote the representations of health conditions in the latent metric space, are recalibrated by the PRS to reduce the bias of naïve prototypes and promote few-shot fault diagnosis. Hence, the trained feature extractor and label predictor are capable of identifying the health condition of machinery in the meta-testing process.

The contributions of the work can be summarized as follows.

(1). To address fault diagnosis with limited data, an innovative end-to-end DAPRN which is made up of a feature extractor, a domain discriminator, and a label predictor is presented. In the training process, the feature extractor learns a representation space by a hybrid training strategy combined with the minimization of few-shot classification loss and the maximization of domain-discriminative loss. In the testing process, the label predictor with recalibrated prototypes can recognize the health conditions of target samples using the generalized meta-knowledge of source diagnostic tasks.
(2). The structure of the feature extractor is appropriately discussed. In addition, to explore whether and how the data capacity and category richness of the source dataset regulate the performance of few-shot fault diagnosis, a series of experiments are designed and carried out. The details of experimental results are analyzed and discussed thoroughly.
(3). To test the validity and superiority of the DAPRN, extensive few-shot fault diagnosis tasks of rolling element bearings and planetary gearboxes under various limited data conditions are conducted. Compared with existing popular FSL methods, in-depth quantitative and qualitative analysis convincingly demonstrates that the proposed method significantly improves the performance of the few-shot fault diagnosis. In addition, ablation studies are implemented to further verify the advance of the proposed method.

The rest of this paper is structured as follows. The background knowledge, including few-shot learning for fault diagnosis, prototypical network based on deep metric learning, and transductive few-shot learning paradigm, is provided in Section 2. Detailed descriptions of the proposed DAPGN are given in Section 3. Section 4 provides an in-depth discussion on multiple few-shot tasks. Finally, Section 5 concludes this research and proposes prospects for future work.

## 2. Background Knowledge

### 2.1. Few-Shot Learning for Fault Diagnosis

**Figure 1:**
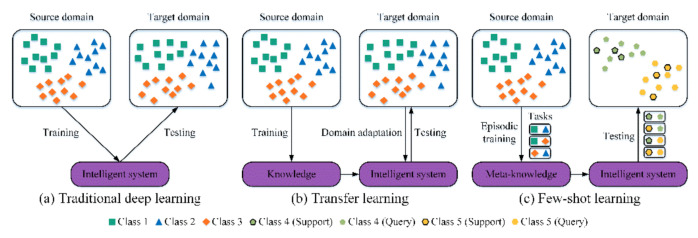
Visualization of (**a**) traditional DL, (**b**) TL, (**c**) FSL.

The workflows of traditional DL, TL, and FSL are demonstrated in Figure 1. Shown in Figure 1a, a supervised fault diagnosis task usually is composed of a training set (source domain) with labeled data for training the traditional DL models and a testing dataset (target domain) for model evaluation. It should be pointed out that the training and testing datasets are drawn from one probability distribution. Shown in Figure 1b, a cross-domain fault diagnosis task consists of a source dataset with labeled data and a target dataset with unlabeled data, and these two datasets are collected from different probability distributions. The TL models, which are trained on the source and target domains employing unsupervised domain adaptation, can correctly recognize samples of the unlabeled target domains. Shown in Figure 1c, the traditional fault diagnosis task is termed a few-shot task when the training dataset contains limited samples with supervised labels. Unlike traditional DL and TL, the source dataset of FSL, whose label space is disjointed from that of the target domain, is named base or auxiliary set with a large amount of labeled data. Note that the training dataset (i.e., support set) with scarce annotated data and testing dataset (i.e., query set) are sampled from the target domain and share the same label space. For clarity, the differences between the above three methods are summarized in Table 1.

Referring to the few-shot classification in computer vision [25,26], the detailed definition of few-shot fault diagnosis is shown as follows. Meta-learning, which is motivated by the learning process of humans and is also known as “learning to learn”, aims to obtain the ability to learn meta-knowledge on higher-level tasks by episodic training. Specifically, there are three datasets in the few-shot fault diagnosis problem: a base set (denoted as B), a support set (denoted as S), and a query set (denoted as Q). For an $N^{T}$-way K-shot M-test few-shot fault diagnosis task (denoted as $T^{T}$) from target domain, the support set $S^{T}$ consists of K samples per class, and the query set $Q^{T}$ consists of M unlabeled examples per class. Note that the support set $S^{T}$ is composed of $M^{T}$ labeled samples $\left\{\left(x_{1}^{T},y_{1}\right),\;\ldots ,\left(x_{M^{T}}^{T},y_{M^{T}}\right)\right\}$ where each $x_{i}^{T}\in {\mathbb{R}}^{D}$ is the D-dimensional representation vector of the original vibration signals and $y_{i}\in \left\{1,2,\ldots ,N^{T}\right\}$ is the corresponding health condition, and $M^{T}$ is equal to $N^{T}\times K$. The base set B consists of $M^{S}$ labeled samples $\left\{\left(x_{1}^{S},y_{1}\right),\ldots ,\left(x_{M^{S}}^{S},y_{M^{S}}\right)\right\}$ in the source domain where each $x_{i}^{S}\in {\mathbb{R}}^{D}$ is raw vibration signal and $y_{i}\in \left\{1,2,\ldots ,N^{S}\right\}$ is the corresponding health condition. It should be pointed out that the $N^{S}$ is equal or greater than $N^{T}$ in practice. During the meta-training phase, the FSL models randomly sample several similar source tasks $T^{S}$s by imitating the target task $T^{T}$. The glamor of episodic meta-learning is to tackle the few-shot fault diagnosis by generalizing from $T^{S}$ based on a known source domain to $T^{T}$ on the target domain. During the meta-testing phase, a trained metric-based model is capable of recognizing the health conditions of the query set $Q^{T}$.

### 2.2. Prototypical Network Based on Metric Learning

The prototypical network (ProtoNet) [27], proposed by Snell et al. in 2017, is becoming one of the most typical and popular metric-based meta-learning methods for FSL applications in various fields. Employing metric learning, ProtoNet can learn a proper metric space in which recognition is conducted by distance measurement between learned prototypes of each class and samples.

**Figure 2:**
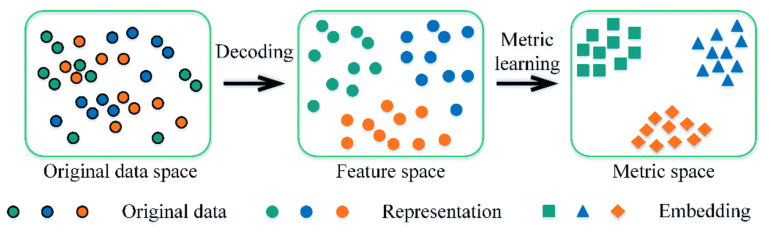
Deep metric learning.

Metric learning, also termed distance metric learning, aims to learn a metric function to measure the similarity among samples automatically. Generally, the metric learning methods usually learn a linear metric function that transforms the samples into representations in a shallow feature space. Owing to the limited ability of metric learning to process complex and massive data, DL-based deep metric learning, one main branch of metric learning, exploits deep architecture to obtain embedded representations for the measurement of similarity [28]. In ProtoNet, the process of deep metric learning is shown in Figure 2. The original data, such as two-dimensional (2D) images and one-dimensional (1D) signals, are fed into one deep decoding network to obtain the representation of the original data in feature space. Then, a metric (e.g., Euclidean, Cosine, Mahalanobis, and Kullback–Leibler) is utilized to make the embeddings per class closer to each other in metric space as the training continues.

**Figure 3:**
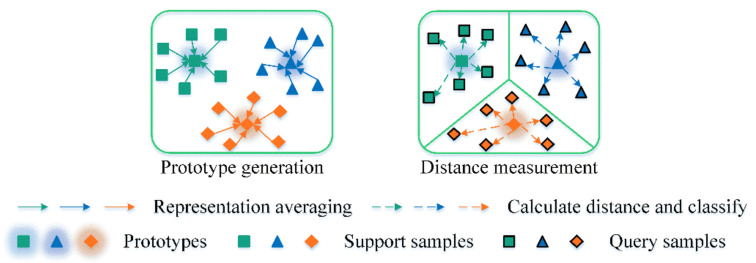
The scheme of ProtoNet.

As shown in Figure 3, the scheme of ProtoNet consists of two main stages: prototype generation and distance measurement. As the key element of ProtoNet, representation averaging is conducted to generate prototypes for each class. The similarity between query samples and learned prototypes is measured, and the query samples can be correctly classified into a certain category.

### 2.3. Transductive Few-Shot Learning Paradigm

With the rapid advance in DL algorithms and computational power, inductive learning-based methods have achieved unprecedented success in traditional supervised tasks [29]. According to the inductive learning paradigm, a machine learning model, which was trained on a training dataset to generalize rules, is applied to predict a testing dataset. In contrast to inductive learning, transductive learning-based methods encountered both training and testing datasets during the training process [30].

Recently, a large body of research has exploited transductive inference in few-shot applications [31,32,33]. In the transductive few-shot learning paradigm, the FSL models will use both the unlabeled samples in the query set and labeled samples in the support set during the meta-training phase, instead of training on support samples in inductive learning-based methods. For instance, Dhillion et al. proposed a transductive few-shot baseline that improves the few-shot classification performance by minimizing the entropy on the predictions of query samples during the meta-training phase [32]. Further, the transductive relation-propagation network fully exploits the relations between the support set and query set by relation propagation [34]. There is a great deal of research indicating that transductive learning-based methods, which serve as an increasingly appealing method to solve few-shot tasks, outperform inductive learning-based methods in many few-shot classification scenarios [29,35]. 

## 3. Proposed Method

### 3.1. The Architecture of Proposed DAPRN

In this work, intelligent fault diagnosis, in which a model trained on a support set S (i.e., training set) with limited labeled data is utilized to predict the health conditions of an unlabeled query set Q (i.e., testing set), is discussed. Paradoxically, a model trained on the support set S with scarce data is usually overfitting and not conducive to effective fault diagnosis on the query set Q in practical engineering. Given the commonality in machine fault diagnosis tasks, such as comparable rolling element bearings installed in different positions or the same position under various operating conditions, it is expected that a somewhat complex few-shot fault diagnosis task can be accomplished if a related labeled dataset with relatively sufficient data, also known as base set B, is available.

**Figure 4:**
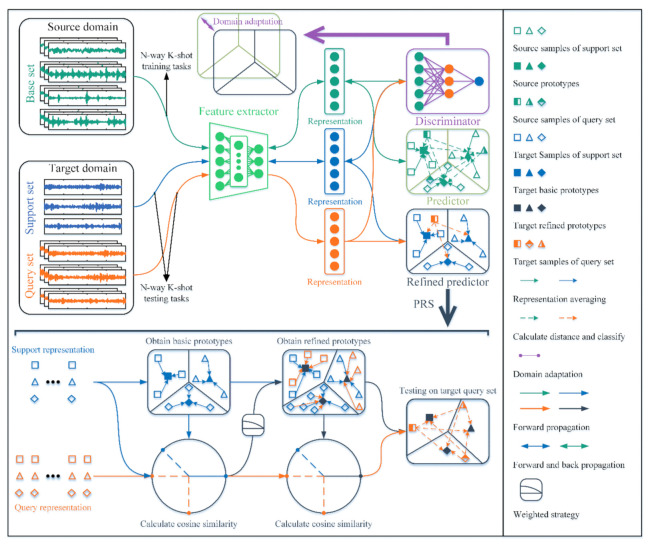
The proposed DAPRN.

The architecture of the proposed end-to-end DAPRN for a few-shot fault diagnosis of machinery is shown in Figure 4. For the fault diagnosis tasks with limited data, the DAPRN is fed with original 1D vibration signals and trained by the episodic training method. It must be noticed that the DAPRN, which is extended from the naïve ProtoNet, is a transductive metric-based meta-learning method. As illustrated in Figure 4, the DAPRN is composed of three main parts, including a feature extractor $f_{FE}$, a domain discriminator $f_{D}$, and a prototype-based label predictor $f_{LP}$. Given the dimension of original vibration signals, the feature extractor $f_{FE}$ is designed as a 1D CNN with powerful feature extraction ability from sequential data. Following [36,37], the feature extractor $f_{FE}$ consists of multiple 1D convolutional and maxpooling layers. More specifically, the convolution operation extracts local temporal features from original vibration signals and the max-pooling operation recognizes the most important features from the output of the last convolutional layer. The domain discriminator $f_{D}$, which is connected with the feature extractor $f_{FE}$, is designed with two fully-connected layers. During the training phase, the flattened representation vectors of source and target domains are input into the $f_{D}$. Due to transductive domain adaptation, the learned metric space of the feature extractor $f_{FE}$ is adapted to enhance the ability of generating more effective task-specific representations in the target domain during the beta-testing phase. The prototype-based label predictor $f_{LP}$ is a module based on cosine distance and prototype recalibration without trainable parameters. It is noted that the refined predictor is the trained predictor $f_{LP}$ with prototype recalibration during the training phase.

### 3.2. Optimization Objective Function

As illustrated in Figure 4, there are two optimization objectives in the training procedure of DAPRN. (1) For the feature extractor $f_{FE}$ with trainable parameters $\theta_{FE}$, minimizing the few-shot classification error $L_{C}$ of the source support-query data guides the $f_{FE}$ to learn an effective metric space for few-shot fault diagnosis tasks. (2) For the domain discriminator $f_{D}$ with trainable parameters $\theta_{D}$, maximizing the domain-discriminative loss $L_{D}$ of data of both domains is capable of achieving marginal distribution alignment, thus further benefiting the task-specific representation extraction in the target domain.

For an N-way K-shot fault diangosis task $T^{T}$, the base set B is composed of a series of annotated subsets $D_{n}^{S}\left(n=1,\ldots ,N^{S}\right)\left(N^{S}\geq N\right)$. At the beginning of each training epoch, N out of $N^{S}$ categories are randomly selected to mimic the $T^{T}$, and each selected subset $D_{n}^{S}$ for category n is chosen with K samples as support set $S_{n}^{S}={\left\{\left(x_{S,i}^{S},y_{S,i}\right)\right\}}_{i=1}^{K}$ randomly and the rest of the subset is the query set with $M_{train}$ samples $Q_{n}^{S}={\left\{\left(x_{Q,i}^{S},y_{Q,i}\right)\right\}}_{i=1}^{M_{train}}$. Hence, several analogous data-structure few-shot tasks $T^{S}$s are obtained in the source domain for the whole training procedure. For convenience, the $X^{S}$ with $M^{S}$ samples and $X^{T}$ with $M^{T}$ samples represent all chosen samples in source and target domains during one training epoch, respectively.

The proposed DAPRN obtains a prototype in the same way as the naïve ProtoNet. Each prototype $P_{n}$, which is an average vector of the embedded representations obtained by the feature extractor $f_{FE}:{\mathbb{R}}^{D}\to {\mathbb{R}}^{H}$, is defined as follows:

(1) $$P_{n}=\frac{1}{K}\underset{x_{S,i}^{S}\in S_{n}^{S}}{\sum }f_{FE}\left(x_{S,i}^{S}\right)$$

Different from the naïve ProtoNet using Euclidean distance for metric learning, cosine similarity has gained popularity in few-shot fault diagnosis of machinery [37]. Given a cosine similarity function $d_{cos}:{\mathbb{R}}^{H}\times {\mathbb{R}}^{H}\to \left[0,\;1\right]$, the label predictor $f_{LP}$ generates a softmax-based probability p over all categories for a sample $x_{Q,i}^{S}$ of query set $Q^{S}$ in the embedding metric space as follows:

(2) $$p\left(y=n|x_{Q,i}^{S}\right)=f_{LP}\left(f_{FE}\left(x_{Q,i}^{S}\right)\right)=\frac{\exp\left(d_{cos}\left(f_{FE}\left(x_{Q,i}^{S}\right),P_{n}\right)\right)}{\sum_{n=1}^{N}exp\left(d_{cos}\left(f_{FE}\left(x_{Q,i}^{S}\right),P_{n}\right)\right)}$$

Based on the probability $p_{m,n}$ of the mth sample for category n in an episode, the standard cross-entropy loss is adopted to minimize the one episodic classification loss $L_{C}$ as follows:

(3) $$L_{C}=\overset{M}{\underset{m=1}{\sum }}\overset{N}{\underset{n=1}{\sum }}\left(-y_{i}log(p_{m,n}\right)-\left(1-y_{i}\right)log\left(1-p_{m,n}\right))$$

As shown in Figure 4, the domain discriminator $f_{D}$ is fed with the embedded representations $f_{FE}\left(x\right)$ with the input being $x\in X^{S}\cup X^{T}$. In this work, the source-domain and target-domain labels are set to 0 and 1, respectively. The output probability $p_{d}$ of the domain discriminator $f_{D}$ is calculated by a softmax layer in the binary cross-entropy loss as follows:

(4) $$p_{d}\left(y=i|x\right)=\frac{\exp\left(f_{D}\left(f_{FE}\left(x\right)\right)\right)}{\sum_{i=0}^{1}exp\left(f_{D}\left(f_{FE}\left(x\right)\right)\right)}$$

As a result, the domain-discriminative loss can be computed as follows:

(5) $$L_{D}=-\frac{1}{M^{S}}\underset{i=1}{\sum^{M^{S}}}\;\log p_{d}\left(y=0|x\in X^{S}\right)-\frac{1}{M^{T}}\underset{i=1}{\sum^{M^{T}}}\;\log p_{d}\left(y=1|x\in X^{T}\right)$$

In the training process, the $L_{C}$ is minimized to obtain a metric space for few-shot fault diagnosis, and the $L_{D}$ is maximized for making two task spaces as similar as possible. To make the training process feasible, a gradient reversal layer (GRL) [38], which is an identity mapping during the forward propagation process and reverses the gradient by multiplying −1 during the backpropagation process, is implemented to connect the feature extractor $f_{FE}$ to the domain discriminator $f_{D}$. Taken altogether, the two optimization objectives of the DAPRN could be summarized as follows:

(6) $$L=L_{C}-\mu L_{D}$$

During the training process, the feature extractor $f_{FE}$ is updated by minimizing the few-shot classification loss $L_{C}$ and maximizing the domain-discriminative loss $L_{D}$ concurrently. Meanwhile, the domain discriminator $f_{D}$ is optimized by minimizing the domain-discriminative loss $L_{D}$. Consequently, the optimization process seeks relatively optimal parameters ${\hat{\theta }}_{FE}$, ${\hat{\theta }}_{D}$ that deliver a saddle point of the overall objective L as follows:

(7) $${\hat{\theta }}_{FE}=\arg\left\{\underset{\theta_{FE}}{min}\;L_{C}\left(\theta_{FE}\right),\underset{\theta_{FE}}{max}\;L_{D}\left(\theta_{FE},{\hat{\theta }}_{D}\right)\right\}$$

(8) $${\hat{\theta }}_{D}=\arg\underset{\theta_{D}}{min}L_{D}\left({\hat{\theta }}_{FE},\theta_{D}\right)$$

Accordingly, the trainable parameters ${\hat{\theta }}_{FE}$ and ${\hat{\theta }}_{D}$ of the DAPRN can be optimized during each training epoch as follows:

(9) $$\theta_{FE}\to \theta_{FE}-𝓁\left(\frac{\partial L_{C}}{\partial \theta_{FE}}-\mu \frac{\partial L_{D}}{\partial \theta_{FE}}\right)$$

(10) $$\theta_{D}\to \theta_{D}-𝓁\frac{\partial L_{D}}{\partial \theta_{D}}$$

where 𝓁 is the learning rate.

When the training procedure is finished, the prototype-based class predictor $f_{LP}$ is capable of recognizing the unlabeled samples in the target domain with a target task-specific metric space generated by the trained feature extractor $f_{FE}$.

### 3.3. Prototype Recalibration Strategy

During the meta-testing procedure, the unlabeled samples of target query set $Q^{T}={\left\{x_{Q,i}^{T}\right\}}_{i=1}^{N\times M}$, where M indicates the number of query samples per category, can be identified by finding the most similar prototype based on the trained feature extractor $f_{FE}$ previously and label predictor $f_{LP}$. However, the naïve prototype, which is determined by such a limited-data regime, is not the one we expected to find in practice. To alleviate the prototype bias caused by the narrow-size target distribution, a PRS is introduced to improve the few-shot fault diagnosis in the meta-testing phase.

Given a target support set $S_{n}^{T}={\left\{\left(x_{S,i}^{T},y_{S,i}\right)\right\}}_{i=1}^{K}$ with K samples for category n, the PRS obtains the naïve prototype $P_{n}$ as follows:

(11) $$P_{n}=\frac{1}{K}\underset{x_{S,i}^{T}\in S_{n}^{T}}{\sum }f_{FE}\left(x_{S,i}^{T}\right)$$

A pseudo-labeling approach, which adds provisional labels for query samples according to prediction confidence, is utilized to obtain a recalibrated prototype in a high-data regime to decrease the bias of naïve prototypes. However, a simple averaging of representation generated from the support set and labeled query set might lead to a worse bias in practice. Therefore, a weighted strategy is carried out to reassign the contribution of the sample from the enhanced support set to the recalibrated prototypes. In detail, top Z samples with the pseudo label being category n are chosen from the query set, then a pseudo-labeled query subset $Q_{pn}^{T}$ is obtained. Specifically, the $w_{i,n}$ represents the contribution of the ith sample from the enhanced support set $S_{n}^{T}\cup Q_{pn}^{T}$ to the recalibrated prototype $P_{n}^{r}$. Then, the recalibrated prototype $P_{n}^{r}$ of category n is computed with reassigned weights $w_{i,n}$ as follows:

(12) $$P_{n}^{r}=\frac{1}{K+Z}\underset{x_{i}^{T}\in S_{n}^{T}\cup Q_{pn}^{T}}{\sum }f_{FE}\left(x_{i}^{T}\right)\times w_{i,n}$$

where $x_{i}^{T}$ indicates a sample from the dataset $S_{n}^{T}\cup Q_{pn}^{T}$. Notably, the weight $w_{i,n}$ is defined by a softmax operation and cosine distance $d_{cos}$ as follows:

(13) $$w_{i,n}=\frac{\exp\left(d_{cos}\left(f_{FE}\left(x_{i}^{T}\right),P_{n}\right)\right)}{\sum_{i=1}^{Z}\exp\left(d_{cos}\left(f_{FE}\left(x_{i}^{T}\right),P_{n}\right)\right)}\left(x_{i}^{T}\in Q_{pn}^{T}\right)$$

In contrast to the naive prototype, the recalibrated prototype, which is determined in a high-data regime, is closer to the optimal prototype in practice.

### 3.4. Transductive Training and Testing Method

For a vanilla metric-based meta-learning method for fault diagnosis, standard episodic training is capable of training a generalized model to novel health conditions in practical engineering. However, this conventional training method is insufficient for the proposed DAPRN. In particular, the training process of the proposed DAPRN is composed of a conventional meta-training procedure and a transductive domain adaptation, whereas the testing process is a PRS-based meta-testing approach. In the training process, the unsupervised domain adaptation via maximization of domain-discriminative loss $L_{D}$ is integrated into the minimization of the few-shot classification loss $L_{C}$. The penalty term of domain-discriminative loss $L_{C}$ is dynamically changed from 1 to 0 and set to $\mu =2-2/\left(1+exp\left(-10\times tp\right)\right)$, and tp denotes the training progress which increases from 0 to 1 gradually. It is worth noting that the domain adaptation using source-domain and target-domain data makes the training procedure a transductive process. In the testing process, a PRS is introduced to solve the basis of the naïve prototype and generate a recalibrated prototype for few-shot fault diagnosis problems.

The Algorithm 1, which includes a transductive training procedure and a few-shot fault diagnosis process based on PRS, is demonstrated as follows.
**Algorithm 1** DAPRN for few-shot fault diagnosis**Transductive training procedure****Input:** For a N-way K-shot fault diagnosis task, base set B in source domain is spilled as a labeled subset $D_{n}^{S}=\left(n=1,\ldots ,N^{S}\right)\left(N^{S}\geq N\right)$. A target-labeled support set $S_{n}^{T}={\left\{\left(x_{S,i}^{T},y_{S,i}\right)\right\}}_{i=1}^{K}$, a target-unlabeled query set $Q_{n}^{T}={\left\{x_{Q,i}^{T}\right\}}_{i=1}^{M}$, number of epoch $n_{E}$, number of episodes $n_{e}$, learning rate 𝓁, penalty term μ **Output:** trained feature extractor $f_{FE}$1. Randomly initialize the parameters of $f_{FE}$, and $f_{D}$ 2. For epoch = 1 to $n_{E}$ 3. Randomly select N out of $N^{S}$ in $D_{n}^{S}$, then obtain $S_{n}^{S}={\left\{\left(x_{S,i}^{S},y_{S,i}\right)\right\}}_{i=1}^{K}$ and $Q_{n}^{S}={\left\{\left(x_{Q,i}^{S},y_{Q,i}\right)\right\}}_{i=1}^{M}$ 4. For episode = 1 to $n_{e}$ 5. Sample few-shot tasks $T^{S}$s from $S_{n}^{S}$ and $Q_{n}^{S}$, then fed them into $f_{FE}$ and $f_{LP}$ 6. Calculate few-shot classification loss $L_{C}$ 7. Sample few-shot tasks $T^{T}$s from $S_{n}^{T}$ and $Q_{n}^{T}$, and fed them into $f_{FE}$ 8. Input representation vectors of source and target domain to $f_{DD}$ 9. Compute the training progress $tp=epoch/n_{E}$ 10. Calculate domain-discriminative loss $L_{D}$ 11. Backpropagation with μ for $L_{D}$ 12. Optimize the parameters of $\theta_{FE}$ and $\theta_{D}$ as follows  $\theta_{FE}\to \theta_{FE}-𝓁\left(\frac{\partial L_{C}}{\partial \theta_{FE}}-\mu \frac{\partial L_{D}}{\partial \theta_{FE}}\right)$ and $\theta_{D}\to \theta_{D}-𝓁\frac{\partial L_{D}}{\partial \theta_{D}}$ 13.   End 14. End
**Few-shot fault diagnosis based on PRS****Input:** A target-labeled support set $S_{n}^{T}={\left\{\left(x_{S,i}^{T},y_{S,i}\right)\right\}}_{i=1}^{K}$, a target-unlabeled query set $Q_{n}^{T}={\left\{x_{Q,i}^{T}\right\}}_{i=1}^{M}$, number of episodes $n_{e}$, number of Z, and trained feature extractor $f_{FE}$ **Output:** Prediction results and average accuracy1. For epoch = 1 to $n_{e}$2.   Sample few-shot tasks $T^{T}$s from $S_{n}^{T}$ and $Q_{n}^{T}$, and fed them into $f_{FE}$ 3. Recalibrate naïve prototypes by PRS as follow $P_{n}^{r}\to PRS\left(Q_{n}^{T},\;P_{n},Z\right)$ 4. Obtain health conditions of $Q_{n}^{T}$ by refined $f_{LP}$ with $P_{n}^{r}$5. End

## 4. Experimental Validation

### 4.1. Dataset Description and Experimental Setup

#### 4.1.1. Bearing Dataset

**Figure 5:**
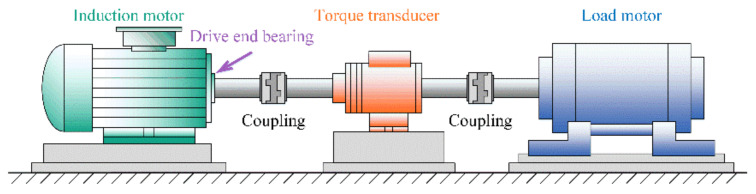
The CWRU bearing test rig.

**Figure 6:**
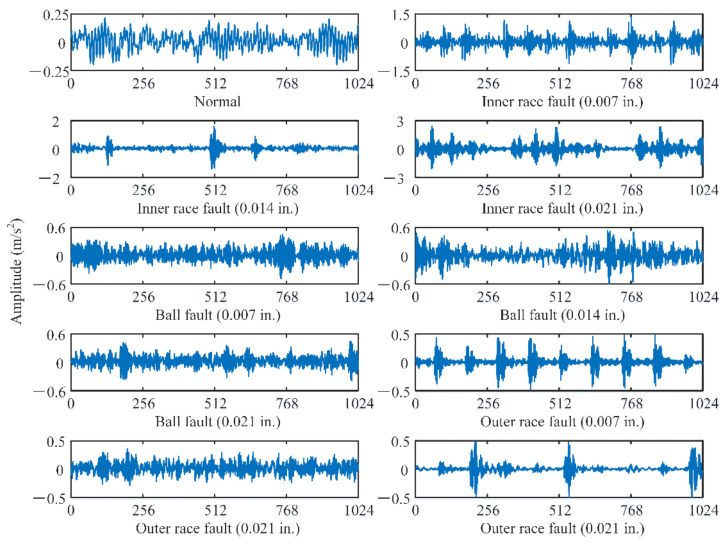
Data sample of bearing under one normal and nine faulty conditions.

To verify the effectiveness and superiority of the proposed DAPRN, the raw vibration signature of bearings, which is collected by the bearing center of Case Western Reserve University (CWRU), is used [39]. As illustrated in Figure 5, the CWRU bearing test rig is composed of three main parts called the induction motor, the torque transducer, and the load motor. Specifically, an accelerometer which is placed at the 12 o’clock position of the driving end bearing of the induction motor is applied to obtain the signature with a sampling frequency of 12 kHz. During experiments, three types of common bearing faults, outer race fault, inner race fault, and ball fault, are simulated by electrical discharge machining. According to the length of artificial damage, three levels of faults (i.e., 0.007 inches, 0.014 inches, and 0.021 inches) were, respectively, seeded on the test bearings. Hence, there are ten health conditions or categories in the CWRU bearings dataset, including one normal health condition and nine types of faults. After that, four different working loads (i.e., 0 hp, 1 hp, 2 hp, and 3 hp) are provided by the load motor to stimulate various working conditions in real industrial scenarios. In our few-shot fault diagnosis, each category contains 200 samples and each sample is a raw vibration signature with 1024 data points. Specifically, the data samples of both normal and faulty conditions are displayed in Figure 6. In addition, the more experimental details of the bearing dataset setting are summarized in Table 2.

#### 4.1.2. Gearbox Dataset

**Figure 7:**
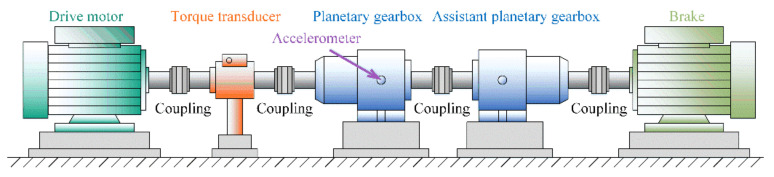
The gearbox test rig.

**Figure 8:**
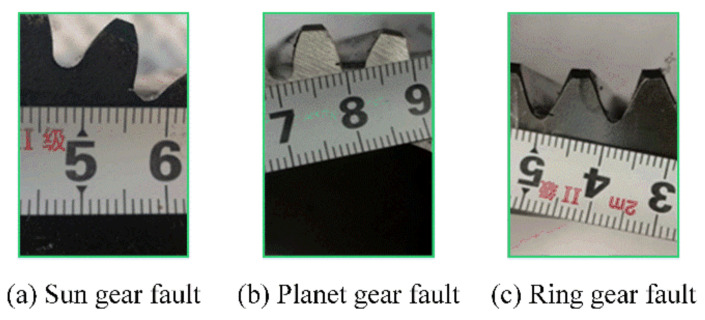
The faults of the gearbox.

Considering that the gearboxes play an important role similar to bearings in modern manufacture, another industrial-use gearbox dataset collected from a two-stage planetary gearbox is utilized to further verify the proposed method on few-shot fault diagnosis tasks. As demonstrated in Figure 7, this gearbox testbed is composed of a variable-speed servo driving motor, a torque transducer, a two-stage planetary gearbox, an assistant planetary gearbox, and a brake. As shown in Figure 7, the accelerometer is placed in the horizontal direction of the second-stage surface of the test gearbox housing to continuously acquire an external vibration signature with a sampling rate of 25.6 kHz. Before experiments, three types of faults with the faulty diameter being 1 mm are simulated on the second stage of the test gearbox, and these faults, including sun gear fault (SF), planetary gear fault (PF), and ring gear fault (RF), are displayed in Figure 8. In this gearbox test rig, each experiment was carried out under three different working conditions. Concretely, the working loads varied between 50 Nm, 150 Nm, and 150 Nm, with a constant rotating speed being 1500 rpm. Similar to the above bearing dataset, each category of the gearbox dataset consisted of 200 samples with 1024 data points. In particular, this dataset’s settings are outlined in Table 3.

#### 4.1.3. Implementation Details

As the indispensable components of mechanical equipment in the production of modern industry, bearings and planetary gearboxes are prone to experience damage accidents in operation. To achieve a few-shot fault diagnosis of the above two key components, the detailed network structures of the feature extractor and domain discriminator for the proposed DAPGN are, respectively, shown in Table 4 and Table 5. It needs to be pointed out that #Param means the number of trainable parameters to the corresponding layer. As demonstrated in Table 4, the feature extractor is composed of one input layer, four 1D convolution-max-pooling blocks (ConvB), and one flattened output layer. Batch normalization (BN) [40], which is used to reduce the internal covariate shift and speed up the network training, is embedded into the proposed network. Furthermore, rectified linear unit (ReLU), one of the most commonly used activation functions, is applied to introduce the nonlinear transformation into the model. Note that the Conv1D, BN-ReLU, and MaxPool1D denote a 1D convolutional layer, a combination of the BN and ReLU, and a 1D max-pooling layer, respectively. In the feature extractor, the stacked four ConvBs play a role in obtaining feature representation from the original vibration signals layer by layer. For instance, in the ConvB1, raw vibration signals whose size is [1 × 1024] are fed into the proposed network. Concretely, the raw vibration signals are input into the Conv1D whose kernel size is 16 × 1 with stride being 2 before the BN layer and ReLU, and the output whose size is [8 × 252] is obtained by the MaxPool1d with kernel size being 2 and stride being 2. In addition, the domain discriminator consists of two FC layers and an ReLU, as shown in Table 5.

Concerning the implementation details of model training, the Adam optimizer [41] with the backpropagation algorithm is adopted to update all trainable parameters of the proposed DAPRN model. During the training procedure, the number of epochs is set to 50. In model training, a decaying learning rate strategy is implemented with the initial learning rate being 0.001 and the decay rate being 0.1. What needs to be pointed out is that the decay epochs are set as 15 and 30, respectively. For the training and testing of the DAPRN model, four commonly used shots in fault diagnosis [42,43], i.e., 1, 3, 5, and 10 shots, are, respectively, set to evaluate the performance of few-shot fault identification, the number of query samples is equal to 200, and the number of episodes, also known as tasks, sampled for training and testing per epoch is set to 100. In the PRS of the meta-testing procedure, the value of Z is set to 20. For clarity, all detailed parameters of the transductive training procedure and meta-testing procedure are summarized in Table 6.

#### 4.1.4. Comparative Methods

To illustrate the efficacy and advantage of the proposed DAPRN, seven popular few-shot learning methods are compared. Notably, all contrastive methods whose feature extractor is built are the same as the feature extractor of the proposed DAPRN for a fair comparison experiment. All details of contrastive methods are illustrated as follows:

1. Baseline [25]: A two-stage Baseline model consists of pretraining on the base set and fine-tuning on the support set. In the pre-training stage, the Baseline model is composed of a feature extractor and a base class classifier. The novel class classifier composed of stacked FC layers is used to replace the base class classifier during the fine-tuning stage.
2. BaselinePlus [25]: The BaselinePlus model is the same as the Baseline model except for the novel classifier. In detail, the classifier, which explicitly reduces the intraclass variations by using a cosine-similarity classification structure, is designed to recognize the health conditions of machinery.
3. SiameseNet [44]: The SiameseNet model, which is composed of a feature extractor and a similarity-measurement module based on a deep neural network, is trained with input being sample pairs of the same or different health conditions of machinery. Note that the sample pairs are randomly selected during the training process.
4. MAML [43]: The MAML model, which is both agnostic to the structure of the feature extractor and loss function, is a bilevel learning paradigm (i.e., inner loop optimization and outer-loop optimization) for meta-knowledge transfer. In detail, the parameters of MAML are quickly updated by inner-loop and outer-loop optimization.
5. ProtoNet [27]: The ProtoNet model, which includes a feature extractor and a Euclidean distance-based label predictor, identifies the health conditions of machinery by using the naïve prototypes of the source domain.
6. MatchingNet [45]: The MatchingNet model, in which the representations of support and query samples are obtained by two independent feature extractors embedded with LSTM, recognizes the health conditions by an attention-based label predictor.
7. RelationNet [46]: The RelationNet model is built on a feature extractor without the last two max-pooling layers and a deep network for metric learning. Specifically, a two-layer CNN is trained for learning a metric space for few-shot fault diagnosis tasks.

The Baseline and BaselinePlus methods, which are both based on a traditional supervised learning paradigm, are trained with original data and tested on the query set. The rest of the few-shot methods are tested by the standard episodic training paradigm. As a consequence, the SiameseNet, MAML, ProtoNet, MatchingNet, RelationNet, and DAPRN report the average accuracy of all episodes during the meta-testing procedure, whereas the Baseline and BaselinePlus report the accuracy during the testing procedure.

### 4.2. Case Study

#### 4.2.1. Situation A: Transfer Learning Scenarios with Limited Data

Transfer learning fault diagnosis scenarios with limited data are widespread in real industrial applications. Based on the few-shot setup, the one-shot fault diagnosis problem across different working conditions is addressed and analyzed by few-shot learning methods. As shown in Table 7, six transfer learning tasks (i.e., AB1, AB2, AB3, AG1, AG2, and AG3) on bearing and gearbox datasets are set to validate the effectiveness and superiority of the proposed DAPRN. The tasks AB1, AB2, and AB3 are 10-way one-shot fault identification of bearing, whereas the tasks AG1, AG2, and AG3 are four-way one-shot fault identification of the planetary gearbox. Notably, each of the six tasks is a close-set transfer learning problem with scarce data and target and source domains sharing the same label space.

The results of the six one-shot fault diagnosis problems are summarized in Table 8, and the bold value denotes the maximum accuracy on this task. Firstly, it can be seen that the proposed DAPRN achieves the highest one-shot fault diagnosis on all bearing and gearbox tasks, with 89.94% accuracy on task AB2 and 99.98% accuracy on task AG1. Secondly, the BaselinePlus method outperforms the Baseline method on all tasks, which reveals that the cosine similarity-based classifier is more effective in transfer learning scenarios with limited data when compared with a network-based classifier. Thirdly, it can be observed that all few-shot methods except for the ProtoNet show better performance on gearbox tasks AG1, AG2, and AG3 when compared with bearing tasks AB1, AB2, and AB3, which indicates that the four-way one-shot problem is substantially easier than the 10-way one-shot problem in practice. A possible explanation for this phenomenon may be the difficulty degree of identifying support-query tasks in the metric space.

#### 4.2.2. Situation B: Cross-Domain Few-Shot Fault Diagnosis

To further prove the validity of DAPRN, another six cross-domain few-shot fault diagnosis tasks of bearing and gearbox datasets are set in this part, as shown in Table 9. In tasks BB1, BB2, BB3, and BG1, the setting of few-shot fault diagnosis is the same as that of the FSL scenarios in computer vision, which means that the label spaces of the source and target domains are completely disjoint with each other. In addition, another two tasks, BG2 and BG3, are carried out to imitate open-set fault diagnosis scenarios with scarce data.

Experimental results of all few-shot fault diagnosis methods are summarized in Table 10. Similarly, the bold values in this table mean the optimal performances of fault diagnosis on each task, and some interesting and enlightening observations can be obtained. Firstly, compared with contrastive methods, the proposed DAPRN with transductive domain adaptation and PRS achieves the best few-shot fault diagnosis performance on all tasks. For instance, the proposed method, respectively, confirms 100.00% accuracy on bearing task BB3 and 98.65% accuracy on gearbox task BG1, which are higher than the other contrastive few-shot approaches by a large margin. Secondly, it can be observed that the dependency of cross-domain few-shot tasks profoundly affects the performance of all few-shot fault diagnosis methods. Particularly, since the tasks BB1 and BB2 are both three-way one-shot fault diagnosis tasks, most few-shot methods on task BB2 achieve dramatically higher performance than those on BB1. These results may be explained by the fact that the proposed DAPRN attempts to identify three outer race faults (i.e., “O1”, “O2”, and “O3”) on task BB1, whereas the base set does not include similar fault types. Thirdly, it is somewhat surprising that the proposed DAPRN achieves a higher accuracy on task BG1 when compared with task BG2, while most few-shot approaches perform significantly better on open-set fault diagnosis task BG2 rather than those on standard few-shot task BG1. There are several likely causes for the differences but it may be related to the task setting between open-set scenarios and few-shot scenarios.

**Figure 9:**
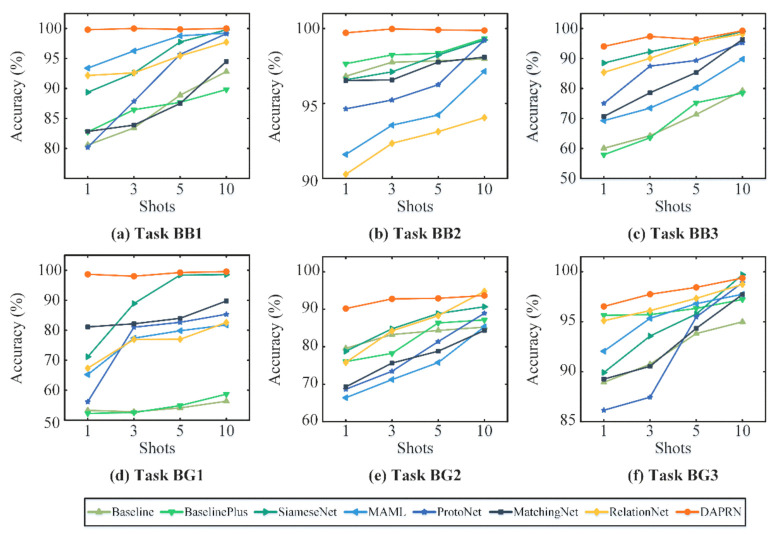
The test accuracy of different shots using all few-shot methods.

More experiments with 3, 5, and 10-shot settings on bearing task BB1 and gearbox task BG1 are carried out for further analysis; the results are summarized in Figure 9 and some interesting findings can be obtained. What stands out in the figure is that the diagnosis accuracy of most contrastive methods increases with the increase of shots on tasks BB1 and BG1. Still, it can be easily observed that no significant differences in the few-shot fault diagnosis performance of the proposed DAPRN are found between the various shots on all tasks. Ulteriorly, the SiameseNet, MAML, ProtoNet, and DAPRN are capable of accomplishing the fault diagnosis of task BB1 with the 10-shot setting at the same level of accuracy. Meanwhile, on 5-shot and 10-shot BG1, the SiameseNet method confirms similar accuracy when compared with the proposed DAPRN. In conclusion, the results in this section indicate that the proposed DAPRN has excellent performance for few-shot fault diagnosis with different shots owing to the transductive domain adaptation and PRS-based meta-testing process.

### 4.3. The Structure of Feature Extractor

In a few-shot fault diagnosis problem, the representation vectors which are generated by the feature extractor have a great impact on the fault classification performance. To illustrate the relationship between the feature network structure and few-shot fault diagnosis, ten trials of the DAPRN with different feature extractors are repeated on two few-shot fault diagnosis tasks, BB1 and BG1, respectively.

**Figure 10:**
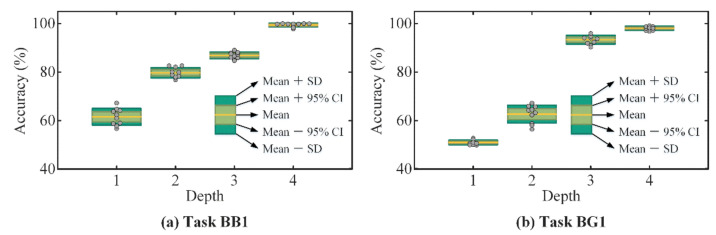
The cross-domain fault diagnosis using the feature extractor with different depths: (**a**) on the three-way one-shot task BB1; (**b**) on the two-way one-shot task BG1.

As illustrated in Table 4, the feature extractor is composed of four ConvBs. The depth of feature extractor to the fault diagnosis on tasks BB1 and BG1 is described in the box in Figure 10, and the statistical characteristics of ten experiments are described using the 95% confidence interval (CI) and standard deviation (SD). More quantitative results and trainable parameters are summarized in Table 11. In Figure 10a, the fault identification accuracy improves and SD reduces with successive increases in depth of the feature extractor on three-way one-shot task BB1, which denotes that the proposed DAPRN tends to be more effective with increasing trainable parameters of the feature extractor. Likewise, it is easily observed that the few-shot diagnostic results on task two-way one-shot BG1 are somewhat counterintuitive, as shown in Figure 10b and Table 11. Surprisingly, the SD on this task is 0.94% when the depth of the feature extractor is 1, which could be attributed to the fact that the proposed DAPRN with a trained one-layer feature extractor misclassifies most of the query samples, including two fault types (i.e., “PF”, and “RF”), as one category. Namely, the one-layer feature extractor with 152 learnable parameters is incapable of performing in few-shot fault diagnosis problems. In summary, the success of the proposed DAPRN could be related to the powerful ability of feature extraction.

### 4.4. Ablation and Parameter Sensitivity Analysis

The DAPRN is composed of a feature extraction module, a domain adaptation module, and a PRS-based meta-testing module. To further illustrate the effectiveness of each module, ablation experiments are carried out on task BB3, and all models for ablation analysis are described as follows.

1. NoDA: This model is designed to examine whether the domain discriminator can improve the generalization ability from the source task to the target task. Hence, a DAPRN model without the domain discriminator is conducted for comparison.
2. NoPR: To describe the effect of the PRS-based meta-testing module, a DAPRN model is implemented where the prototype calibration is cut off. It should be pointed out that the NoPR model is trained the same as the DAPRN model and tested without PRS.

**Figure 11:**
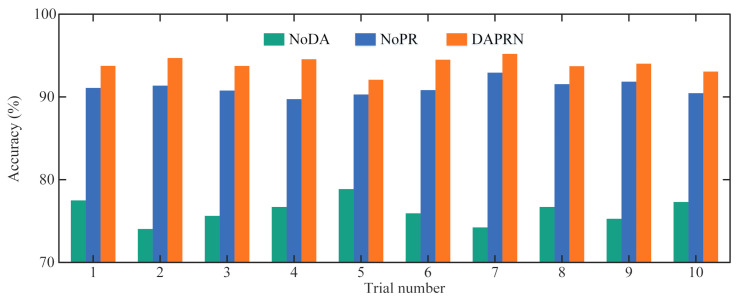
Detailed testing accuracy of ten trials using various models on task BB3.

**Figure 12:**
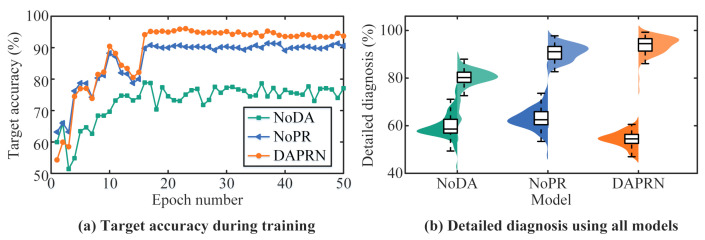
The training process of all models: (**a**) target accuracy during transductive training process; (**b**) detailed diagnosis of one episode: left (epoch = 1), right (epoch = 50).

**Figure 13:**
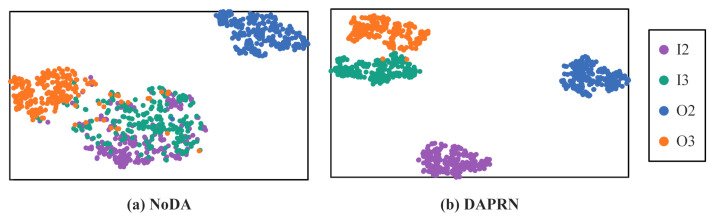
The visualization of target representation space.

The detailed results of ten trials using different models are illustrated in Figure 11. This figure shows that the proposed DAPRN has a better few-shot performance than the NoDA and NoPR, indicating that transductive domain adaption and PRS are both beneficial to improve fault diagnosis with limited data. Meanwhile, all NoDA models perform poorly compared to NoPR models, which shows that the domain discriminator plays a more vital role in few-shot fault diagnosis problems when compared with the prototype recalibration. For further analysis, detailed information on one trial for three models is detailed in Figure 12. As shown in Figure 12a, the diagnostic accuracy of all models converges to a certain degree and the DAPRN confirms the best accuracy when the training process is over. Furthermore, it can be observed that the diagnostic performance of DAPRN is lower than that of NoPR in the early stages of the training process and higher than that of NoPR in the late stages of the training process, implying that the diagnostic performance is only enhanced when the trained label predictor is sufficiently effective. The detailed diagnostic results of one episode at the beginning and end of the transductive training process are shown in Figure 12b, and the kernel density estimation method is applied to fit the probability distribution of detailed results. As can be seen from Figure 12b, the trained DAPRN is capable of outperforming other models even if the diagnostic accuracy of the initial stage is worse than others. In addition to quantitative analysis, the t-distributed stochastic neighbor embedding (t-SNE) [38] is employed for qualitative analysis intuitively in Figure 13. Particularly, the outputs of the feature extractor (i.e., representation vectors) of the NoDA and DAPRN are mapped into a two-dimensional space for clear visualization, respectively. In this figure, the DAPRN gathers the representations of each health condition (i.e., I2, I3, O2, and O3) better than the NoDA, which means that the label predictor of DAPRN has more potential for few-shot fault diagnosis via transductive domain adaptation.

**Figure 14:**
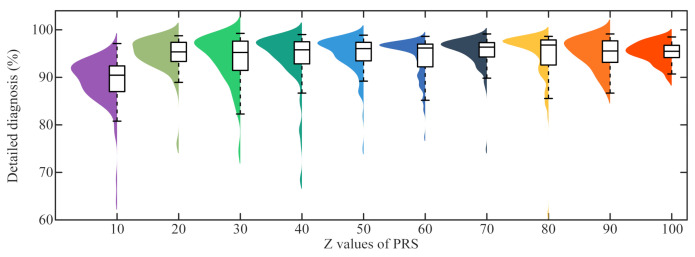
The sensitivity of diagnostic accuracy of the DAPRN to the Z values of PRS.

In practical engineering, the hyperparameter Z of PRS of the proposed DAPRN will impact the few-shot fault diagnosis distinctly. For further analysis, the sensitivity experiment on task BB3 was carried out ten times, and the box Figure 14 demonstrates the few-shot fault diagnosis for the hyperparameter Z of PRS. In detail, it can be found that the performance of the proposed DAPRN with Z being 10 is lower than others on task BB3. Moreover, the performance of the DAPRN nearly reaches a plateau and confirms around 94% accuracy when Z values of PRS are more than 20. Consequently, none of these differences in the few-shot fault diagnosis of DAPRN are statistically significant. This finding was unexpected and suggests that blindly increasing the samples of the query set for PRS does not bring significant benefits for few-shot fault diagnosis tasks. A possible explanation for this might be that the recalibrated prototypes for each health condition are limited by the representations of the feature extractor. In other words, with successive increases in the Z values, the enhancement gained by PRS is not capable of continuing to increase due to the representation space with imperfect feature aggregation. 

## 5. Conclusions

To achieve the issue of few-shot fault diagnosis in real industrial scenarios, an innovative domain-adaptive prototype-recalibrated network (DAPRN) based on transductive domain adaptation and prototype recalibration strategy (PRS) is proposed, comprising a feature extractor, a domain discriminator, and a label predictor. Compared with popular few-shot fault diagnosis methods, the DAPRN jointly conducts source few-shot task classification and domain adaptation across domains, and the meta-knowledge of source diagnostic tasks is generalized to the target tasks through domain adaptation in the transductive training stage effectively. To reduce the bias of the naïve prototypes, the PRS is implemented to promote the few-shot fault diagnosis in the meta-testing stage. Extensive experiments on bearing and gearbox datasets demonstrate outstanding fault diagnosis performance under omnifarious limited data conditions. The quantitative and qualitative analysis convincingly reveals that the proposed method outperforms other few-shot fault diagnosis methods. In terms of real industrial application, the proposed method is promising to address more challenging fault diagnosis scenarios with limited data and promote practical fault identification problems of machinery. 

The most important limitations of this study lie in the fact that the proposed method can only perform offline predictions. In addition, a high-quality annotated related dataset is required. In the future, not only meta-knowledge generalization across different machines but also few-shot fault diagnosis with an insufficient base dataset should be undertaken to solve more fault identification tasks in real industrial scenarios.

## Figures and Tables

**Table 1:** The differences between the three methods (i.e., traditional DL, TL, and FSL).

Methods	Domains	Source and Target Task	Source Label	Target Label
Traditional DL	Same	Same	Available	Available
TL	Different	Same or related	Available	Unavailable
FSL	Same or different	Different	Available	Limited labels

**Table 2:** Bearing dataset settings under four working loads (0 hp, 1 hp, 2 hp, and 3 hp).

Bearing Health Condition	Fault Diameter (in.)	The Number of Samples	Category Abbreviation
Normal	/	200	N
Inner race fault	0.007	200	I1
	0.014	200	I2
	0.021	200	I3
Ball fault	0.007	200	B1
	0.014	200	B2
	0.021	200	B3
Outer race fault	0.007	200	O1
	0.014	200	O2
	0.021	200	O3

**Table 3:** Gearbox dataset settings under three working loads (50 Nm, 150 Nm, and 250 Nm).

Gearbox Health Condition	Fault Diameter (mm)	The Number of Samples	Category Abbreviation
Normal	/	200	Nor
Sun gear fault	1	200	SF
Planet gear fault	1	200	PF
Ring gear fault	1	200	RF

**Table 4:** Structure of the feature extractor.

Symbol	Layer	Output Size	Parameter	#Param
Input	Input	[1 × 1024]	/	/
Layer1 (ConvB1)	Conv1D	[8 × 505]	kernel size = 16, stride = 2	136
	BN-ReLU	[8 × 505]	/	16
	MaxPool1D	[8 × 252]	kernel size = 2, stride = 2	/
Layer2 (ConvB2)	Conv1D	[16 × 125]	kernel size = 3, stride = 2	400
	BN-ReLU	[16 × 125]	/	32
	MaxPool1D	[16 × 62]	kernel size = 2, stride = 2	/
Layer3 (ConvB3)	Conv1D	[32 × 30]	kernel size = 3, stride = 2	1568
	BN-ReLU	[32 × 30]	/	64
	MaxPool1D	[32 × 15]	kernel size = 2, stride = 2	/
Layer4 (ConvB4)	Conv1D	[64 × 7]	kernel size = 3, stride = 2	6208
	BN-ReLU	[64 × 7]	/	128
	MaxPool1D	[64 × 3]	kernel size = 2, stride = 2	/
Output	Flatten	192	/	/

**Table 5:** Structure of the domain discriminator.

Symbol	Layer	Output Size	Parameter	#Param
Layer1	FC1	64	In_features = 192, out_features = 64	12,352
Layer2	ReLU	64	/	/
Layer3	FC2	2	In_features = 64, out_features = 2	130

**Table 6:** Parameter settings of the transductive training method.

Parameters	Value	Parameters	Value
Learning rate	0.001	Support samples per category (K)	1, 3, 5, or 10
Decay rate	0.1	Query samples per category (M)	200
Maximum epochs	50	Episodes of source and target tasks	100
Decay epoch	15, 30	Z of PRS	20

**Table 7:** Transfer learning scenarios of bearing and gearbox datasets.

Dataset	Source Domain	Target Domain	Source Categories	Target Categories	Task Abbr.
Bearing	0 hp	1 hp	All health conditions	All health conditions	AB1
Bearing	1 hp	2 hp	All health conditions	All health conditions	AB2
Bearing	2 hp	3 hp	All health conditions	All health conditions	AB3
Gearbox	50 Nm	150 Nm	All health conditions	All health conditions	AG1
Gearbox	150 Nm	250 Nm	All health conditions	All health conditions	AG2
Gearbox	250 Nm	50 Nm	All health conditions	All health conditions	AG3

**Table 8:** Accuracy on one-shot fault diagnosis of transfer learning scenarios.

Methods	AB1	AB2	AB3	AB4	AB5	AB6
Baseline	81.06%	81.01%	81.41%	93.72%	92.46%	90.08%
BaselinePlus	86.03%	86.43%	85.28%	96.23%	96.11%	95.98%
SiameseNet	79.71%	80.34%	79.87%	97.15%	95.71%	96.39%
MAML	78.99%	80.14%	82.37%	95.73%	95.37%	95.13%
ProtoNet	83.86%	83.11%	84.28%	82.74%	82.25%	81.61%
MatchingNet	82.64%	85.95%	84.79%	87.22%	88.17%	85.45%
RelationNet	81.95%	83.79%	83.67%	88.05%	87.12%	87.76%
DAPRN	**88.16%**	**89.94%**	**89.19%**	**99.98%**	**98.94%**	**98.51%**

The highest accuracy is highlighted in bold.

**Table 9:** Cross-domain few-shot scenarios of bearing and gearbox datasets.

Dataset	Source Domain	Target Domain	Source Categories	Target Categories	Task Abbr.
Bearing	0 hp	1 hp	N, I1, I2, I3, B1, B2, B3	O1, O2, O3	BB1
Bearing	1 hp	2 hp	N, I2, I3, B2, B3, O2, O3	I1, O1, B1	BB2
Bearing	2 hp	3 hp	N, I1, B1, B2, B3, O1	I2, I3, O2, O3	BB3
Gearbox	50 Nm	150 Nm	Nor, SF	PF, RF	BG1
Gearbox	150 Nm	250 Nm	Nor, SF, PF	Nor, PF, RF	BG2
Gearbox	250 Nm	50 Nm	Nor, SF, PF, RF	SF, PF, RF	BG3

**Table 10:** Accuracy on one-shot fault diagnosis of transfer learning scenarios.

Methods	AB1	AB2	AB3	AB4	AB5	AB6
Baseline	80.57%	96.82%	60.05%	53.27%	79.56%	88.94%
BaselinePlus	82.75%	97.65%	57.91%	52.26%	76.05%	95.64%
SiameseNet	89.37%	96.58%	88.49%	71.14%	78.80%	89.92%
MAML	93.39%	91.60%	69.28%	65.21%	66.40%	92.05%
ProtoNet	80.19%	94.64%	75.01%	56.14%	68.66%	86.15%
MatchingNet	82.84%	96.53%	70.68%	81.10%	69.29%	89.26%
RelationNet	92.16%	90.27%	85.37%	67.33%	75.80%	95.11%
DAPRN	**99.80%**	**99.71%**	**94.02%**	**98.65%**	**90.19%**	**96.53%**

The highest accuracy is highlighted in bold.

**Table 11:** The detailed one-shot cross-domain fault diagnosis result (average accuracy and standard deviation) and #Param using the feature extractor with different depths.

Depth of Feature Extractor	1	2	3	4
Accuracy on task BB1	61.56% ± 3.47%	79.71% ± 2.13%	86.87% ± 1.36%	99.44% ± 0.79%
Accuracy on task BG1	51.00% ± 0.94%	62.68% ± 3.69%	93.35% ± 1.87%	98.09% ± 0.80%
#Param	152	584	2216	8552

## Data Availability

Not applicable.
